# Impact of a New York City supportive housing program on Medicaid expenditure patterns among people with serious mental illness and chronic homelessness

**DOI:** 10.1186/s12913-017-2816-9

**Published:** 2018-01-10

**Authors:** Sungwoo Lim, Qi Gao, Elsa Stazesky, Tejinder P. Singh, Tiffany G. Harris, Amber Levanon Seligson

**Affiliations:** 1 Bureau of Epidemiology Services, Division of Epidemiology, New York City Department of Health and Mental Hygiene, New York, NY USA; 2 Albert Einstein School of Medicine, New York, NY USA; 3 HRA Customized Assistance Services, New York, NY USA; 4 ICAP, Mailman School of Public Health, Columbia University, New York, NY USA

**Keywords:** Medicaid, Housing, Homeless

## Abstract

**Background:**

A rapid increase of Medicaid expenditures has been a serious concern, and housing stability has been discussed as a means to reduce Medicaid costs. A program evaluation of a New York City supportive housing program has assessed the association between supportive housing tenancy and Medicaid savings among New York City housing program applicants with serious mental illness and chronic homelessness or dual diagnoses of mental illness and substance use disorder, stratified by distinctive Medicaid expenditure patterns.

**Methods:**

The evaluation used matched data from administrative records for 2827 people. Sequence analysis identified 6 Medicaid expenditure patterns during 2 years prior to baseline among people placed in the program (*n* = 737) and people eligible but not placed (*n* = 2090), including very low Medicaid coverage, increasing Medicaid expenditure, low, middle, high, and very high Medicaid expenditure patterns. We assessed the impact of the program on Medicaid costs for 2 years post-baseline via propensity score matching and bootstrapping.

**Results:**

The housing program was associated with Medicaid savings during 2 years post-baseline (−$9526, 95% CI = −$19,038 to -$2003). Stratified by Medicaid expenditure patterns, Medicaid savings were found among those with very low Medicaid coverage (−$15,694, 95% CI = −$35,926 to -$7983), increasing Medicaid expenditures (−$9020, 95% CI = −$26,753 to -$1705), and high Medicaid expenditure patterns (−$14,450, 95% CI = −$38,232 to -$4454). Savings were largely driven by shorter psychiatric hospitalizations in the post-baseline period among those placed.

**Conclusions:**

The supportive housing program was associated with Medicaid savings, particularly for individuals with very low Medicaid coverage, increasing Medicaid expenditures, and high Medicaid expenditures pre-baseline.

**Electronic supplementary material:**

The online version of this article (10.1186/s12913-017-2816-9) contains supplementary material, which is available to authorized users.

## Background

Medicaid is a public health insurance program in the United States (US) that provides comprehensive healthcare services to vulnerable populations including individuals with disabilities, older adults, and low-income families [1]. Several studies have demonstrated that almost 50% of total US Medicaid expenditures are driven by only about 4% of Medicaid enrollees who are characterized by being elderly and/or having non-stable housing; having mental illness, chronic medical conditions, or substance use disorders; and being in long-term institutional care [2]. As Medicaid expenditures have rapidly increased from $201 billion in 2000 to $408 billion in 2011 [3], there has been growing interest in the reduction of Medicaid costs by improving housing stability [4, 5]. In particular, the “housing first model” where unstably-housed individuals are placed into permanent housing without requiring adherence to substance use treatment or other services has been proposed as an intervention for health-promoting behaviors and improved healthcare access [6, 7]. These benefits, in turn, are thought to decrease the likelihood of high-cost medical events such as emergency department visits and hospitalizations [5, 8–10].

A recent review of US and Canadian studies found that the housing first model for homeless individuals with serious mental illness has led to decreases in emergency department (ED) visits and hospitalizations [11]. However, among unstably-housed individuals who incur high medical costs due to frequent ED visits or hospitalizations, no studies have identified high users based on duration and sequencing of medical expenditures. Given that extremely high Medicaid costs are often due to only a few hospitalization events, using aggregated Medicaid costs as a threshold for high Medicaid users (e.g., 75th percentile of 1-year total Medicaid costs among Medicaid enrollees) may not identify individuals with consistently high Medicaid costs. In a program evaluation of supportive housing in New York City (NYC), we sought to determine whether there was an association between supportive housing tenancy and Medicaid savings among those with serious mental illness and chronic homelessness or dual diagnoses of mental illness and substance use when stratified by distinctive Medicaid expenditure patterns. We employed group-based trajectory modeling to identify groups of individuals who shared similar trajectories of monthly Medicaid expenditures during the 2-year pre-housing period.

## Methods

### Setting and sample

The population in this evaluation was composed of individuals aged 18 years or older who were eligible for a NYC supportive housing program because they met one the 2 following conditions: 1) chronic homelessness and serious mental illness or, 2) dual diagnosis of mental illness and a substance use disorder. This is one of 9 population groups in a NYC supportive housing program launched by NYC and New York State governments in 2007 to create 9000 supportive housing units for people who were homeless or at risk of homelessness [12]. The program followed the housing first model with housing placement not being contingent on adhering to treatment or services. Chronic homelessness is determined if individuals had to have a record of either 1) staying at least 2 out of the last 4 years in a homeless shelter or living on the street or 2) being disabled and spending at least one of the last 2 years in shelter or living on the street. To qualify as having a mental illness condition, they submitted comprehensive psychiatric evaluations signed by psychiatrists or nurse practitioners conducted within 6 months prior to the application date. This clinical information (i.e., Axis I and Axis II according to the Diagnostic and Statistical Manual of Mental Disorders IV criteria and a comprehensive psychosocial summary) in the housing application was independently assessed and verified by clinical staff from the NYC Human Resources Administration. We matched multiple administrative records to evaluate the program’s impact on utilization of government services and benefits Data included utilization of government housing, jails, homeless shelters, New York State psychiatric facilities, Medicaid claims, and food stamps. These data were provided by the NYC Human Resources Administration’s Customized Assistance Services and HIV/AIDS Services Administration, the NYC Department of Health and Mental Hygiene, the NYC Department of Homeless Services, the NYC Department of Correction, and the New York State Office of Mental Health. Data matching was performed deterministically between service data (Medicaid, State psychiatric facilities, food stamps, some types of subsidized housing) and the NYC supportive housing program data using identifiers (first and last name, birth date, and social security number). For all the other data, we performed probabilistic matching using first, middle, and last names, birth date, social security number, sex, and address (when available) via QualityStage software (IBM Corporation, Armonk, New York) [13]. According to an independent human review of sample cases for the probabilistic match, matching performance was acceptable (sensitivity: 93%, specificity: 97%).

In this evaluation, we included individuals who were eligible for the program during 2007–10. Housing or program directors received multiple eligible applicants per 1 vacancy and determined a placement decision after interviewing a maximum of 3 candidates. We categorized these individuals into 2 groups: 1) applicants who were placed in the program for more than 7 days (“placed”) and 2) applicants who were not placed in the program during 2 years after eligibility (“unplaced”), and were not placed in any other government-subsidized housing tracked by the evaluation during the first 6 months after eligibility (i.e., program eligibility period) to ensure lack of exposure to a supportive housing program during the initial treatment assignment. We excluded those who were housed in other government-subsidized housing programs immediately before becoming placed in the program (no gap between move-out and move-in dates; *n* = 20) because these continuous housing experiences might have confounded changes in outcomes associated with the program. Baseline (i.e., index date) was defined as the first placement date (for the placed group) or the first eligibility date (for the unplaced group). For each individual, we examined the 2-year period prior to baseline and 2-year period post baseline. For example, for those who became eligible for the program on December 31, 2010, the evaluation period was January 1, 2008 through December 31, 2012. For placed people, a median of 95 days (51 days on 25th percentile, 208 days on 75th percentile) passed between the first eligibility date and the first move-in date. This gap between the eligibility and placement dates reflected administrative processes to finalize placement (e.g., documentation, service arrangement, and entitlement paperwork).

For the analysis, we retained placed applicants in the placed group even if they left the program, and unplaced applicants were included in the unplaced group even if they were placed in any other government-subsidized housing after the first 6-month period. On average the placed group stayed in the program for 661 days (162 on standard deviation, 730 on 25th percentile, 730 days on median, 730 days on 75th percentile) during the 2 years after placement. The final dataset contained 2827 people. This sample selection process was illustrated in Additional file 1. The NYC Department of Health and Mental Hygiene Institutional Review Board determined that this was a program evaluation activity and non-human subject research, and therefore did not fall under the purview of the Institutional Review Board.

### Variables

In this program evaluation, the exposure variable was an indicator of supportive housing placement (defined above). The outcomes were aggregated Medicaid costs during 2 years after baseline, adjusted for medical inflation to 2012 dollars. The total Medicaid costs were further divided into costs from 1) outpatient care, 2) inpatient care, 3) emergency department visits, and 4) prescription drugs. The remaining costs (“other costs”) mainly consisted of those from home health agencies and personal care, and residential care. Capitation payments were classified in the other costs category and combined with fee-for-service costs because Medicaid savings in this evaluation were assessed using the cost-to-government perspective (i.e., government payments for Medicaid services) rather than the societal cost perspective. Because capitation payments did not provide information about costs associated with specific use of services, service-specific costs in the remaining categories might have been underestimated; yet this potential bias is likely small because behavioral health-related claims, which represented most claims in this population, were carved out and reimbursed via a fee-for-service mechanism during the evaluation period.

As an additional outcome, we calculated the percent of individuals who participated in managed care for at least 6 months during the 2 year pre- or 2 year post-baseline because pre- and post-changes in managed care enrollment by placement status, which is a cost containment effort, could help explain changes in Medicaid costs associated with the housing program.

Lastly, to describe and account for differences between placed and unplaced people, we included covariates that captured the use of government services and benefits during 2 years prior to baseline using the other administrative data sources and monthly Medicaid coverage dates during 2 years prior to baseline and post-baseline using Medicaid data. To construct the Medicaid expenditure patterns via sequence analysis, we calculated monthly Medicaid expenditures during 2 years prior to baseline. We also obtained demographic and clinical descriptors at the time of program application using the NYC supportive housing application data (Additional file 1).

### Statistical analyses

**Fig. 1:**
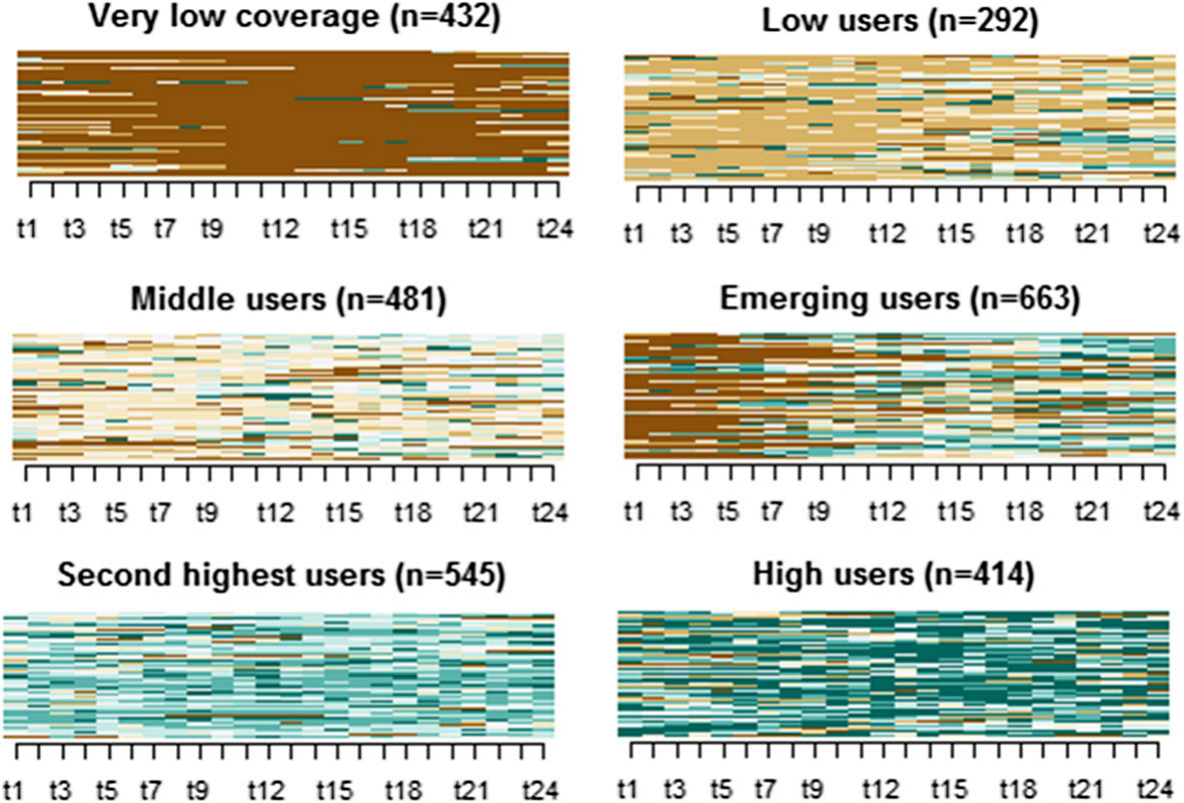
Six Trajectory groups of Medicaid users during 2 years prior to baseline in a New York City supportive housing program. This figure illustrates how individuals are grouped according to similarities in trajectories of monthly Medicaid expenditures over 2 years prior to baseline. No coverage (*dark brown color*); 0 cost (*brown color*); <20th % (*light brown color*); 20th % ~40th % (*white color*); 40th % ~60th % (*light blue color*); 60th % ~80th % (*blue color*); >80th % (*dark blue color*). Each horizontal line in the y axis represents an individual-level sequence of monthly Medicaid cost categories during 2 years prior to baseline. The x axis represents each month during two years prior to baseline. These individual-level sequences were stacked together and divided into 6 distinct clusters (or plots) based on their similarities. The height of the original plot was proportional to the number of individuals in each cluster, but then adjusted to the same size to more clearly show color patterns. NYC Department of Homeless Services, NYC Department of Correction, NYC Department of Health and Mental Hygiene, NYC Human Resources Administration’s Customized Assistance Services and HIV/AIDS Services Administration, and New York State Office of Mental Health

We performed sequence analysis to identify distinctive patterns of Medicaid expenditures during the 2 years prior to baseline among the placed and unplaced groups combined. Sequence analysis is a method that identifies mutually exclusive groups of individuals who share similar trajectories of events (in this analysis monthly Medicaid costs) [14]. This method compares individuals’ duration and sequencing of events among all possible pairs and determines unique temporal patterns [14, 15]. The resulting temporal grouping can be more instructive than grouping based on conventional aggregated measures (e.g., high Medicaid users based on 2-year aggregated Medicaid costs) when there are multiple types of events (e.g., no Medicaid coverage, zero Medicaid cost) and costly outlying events (e.g., psychiatric hospitalizations) [15]. The utility of sequence analysis in health services research has been demonstrated by recent findings of prenatal care trajectories among French population [16] and sequences of clinical instabilities such as fever and hypotension among United States patients who were hospitalized due to community-acquired pneumonia [17]. We first categorized monthly Medicaid costs for each month during the 2-year pre-baseline period using a ranking: 0 = no Medicaid coverage; 1 = $0; 2 = $1–$292; 3 = $293–$690; 4 = $691–$1379; 5 = $1380–$2898; 6 = ≥$2899. Ranks 2 through 6 were based on the quintiles for all non-zero monthly costs. To determine whether zero cost was due to no coverage (rank 0) or no utilization (rank 1), we examined Medicaid coverage in each month when there were zero costs during the 2-year period, and assigned the rank 1 to the month if individuals were on Medicaid; if not, we assigned the rank 0. We converted Medicaid costs into these 7 categories using this ranking approach because there was a high percentage of individuals with no costs and a few individuals who had extremely high expenditures. In Fig. 1, individual-level sequences of monthly Medicaid (categorized by no coverage, zero, and quintile non-zero monthly Medicaid costs and presented using different colors) were represented as horizontal lines. For example, if a line is dark brown-colored up to the first year (= t12) and subsequently light blue-colored for the second year, it represents an individual who initially did not have Medicaid coverage and then consistently incurred small Medicaid costs. A line that does not change over time represents a time period when there is no change in Medicaid costs. Then we adopted the optimal matching algorithm to determine the extent of dissimilarity between pairs of 2-year sequences of monthly Medicaid cost ranks [14]. We translated pair-wise differences of sequences into weights based on transition probabilities, created a distance matrix by applying this procedure to all possible pairs, and then conducted a hierarchical cluster analysis with the Ward method [14, 18]. In other words, individual-level expenditure sequences were stacked together based on their similarities. We found that the cluster analysis identified 6 non-overlapping groups of individuals who shared similar trajectories of monthly Medicaid expenditures over 2 years prior to baseline. By examining these trajectories, we labeled each group to conceptualize distinct patterns of Medicaid expenditures among the NYC supportive housing applicants.

We used propensity score matching to minimize baseline differences between the placed and unplaced groups [19]. For each Medicaid expenditure pattern separately, we ran a logistic regression model to predict the probabilities of placement (i.e., propensity scores) using characteristics of program applicants prior to baseline or at baseline as the independent variables (Additional file 2). In the final propensity score models, we excluded variables with greatly inflated standard errors because these were interpreted as evidence of multicollinearity (Additional file 2). Then, we carried out optimal full matching, which yielded matched sets of varying numbers of individuals from both placed and unplaced groups [20]. In this propensity score matching method, differences between placed and unplaced groups were achieved by using varying matching ratios (e.g., 1:1, 1:N, N:1, and N:N), which is more efficient than the conventional 1:1 nearest neighbor matching [21]. Another advantage of this optimal full matching was it retained all sample members, which was important for this program evaluation because we were interested in the impact of the housing program among all eligible applicants [21]. We assessed the extent to which the propensity score matching reduced observed differences between placed and unplaced individuals by observing the standardized difference in average covariate values before and after matching [22]. Specifically, we calculated the standardized absolute difference by dividing absolute difference in a covariate value between placed and unplaced people (continuous covariate: difference in average scores; categorical covariate: difference of proportion in each category) by pooled standard errors via a variance formula for either continuous or categorical variables. These estimated differences across most covariates and in each of the Medicaid expenditure patterns substantially decreased after the propensity score matching, suggesting that the propensity score matching performed well in minimizing the observed differences (Additional file 3). For each Medicaid expenditure pattern, we calculated mean differences in Medicaid costs between placed and unplaced groups during 2 years post-baseline using standardization, In this technique the mean cost differences were calculated per matched set, and proportionally weighted according to the size of the matched set relative to the total sample size, which allowed accounting for propensity score matching in mean cost difference estimates. To perform statistical tests for the mean differences between the 2 groups, we used 10,000 bootstrap replicates, which is a non-parametric variance estimation approach to address the violation of the normal distribution assumption [22]. To incorporate propensity score matching into this estimation of 95% confidence interval (CI), in each bootstrap replicate, we performed propensity score matching and calculated the mean cost differences using standardization. We corrected bootstrapping distributions using bias and acceleration statistics to address potential bias in point estimates and account for varying variances, which could result from random replications [23]. The percent of the managed care participation, which was a secondary outcome, was compared between placed and unplaced people per each expenditure group using conditional logistic regression that accounted for propensity score matching.

We performed independent t-tests for continuous variables and chi-square tests for categorical variable to test whether placed people were different from unplaced people in baseline or pre-baseline characteristics before and after propensity score matching. In addition, we described baseline characteristics and pre-baseline Medicaid utilization (i.e., Medicaid costs due to outpatient care, inpatient care, emergency department visits, prescription drugs, and other uses) by 6 Medicaid expenditure groups. Similar to tests for difference between placed and unplaced people, we performed independent t-tests for continuous variables and chi-square tests for categorical variables as we tested differences across Medicaid expenditure groups. Statistical significance for these tests was established using 2-tailed *p* < 0.05. For post-baseline mean comparisons, statistical significance was established using 95% confidence intervals via bootstrapping. Sequence analysis, optimal full matching, and bootstrapping were performed using TraMineR, cluster, optmatch, and boot packages in R 3.3.1 software (Vienna, Austria) [24]. All other analyses were performed using SAS 9.4 software (Cary, NC) [25].

## Results

Table 1 shows that a majority of the eligible applicants were non-Hispanic Black or Hispanic males and did not need assistance with activities of daily living (i.e., capacity of living independently). Sixty four percent were aged to 35 years to 54 years at the time of the application. About 50% were diagnosed with substance use disorders, while 74% reported not currently using substances. Baseline demographic and behavioral characteristics were in general similar between the placed and unplaced groups, and differences in particular characteristics (e.g., higher likelihood of being capable of living independently among the placed group) substantially decreased after propensity score matching (Table 1).

**Table 1:** Selected baseline demographic, clinical, and service utilization characteristics at the time of application to the New York City supportive housing program by placement status

		Before propensity score matching			After propensity score matching		
	Total	Placed	Unplaced	*p*-values	Placed	Unplaced	*p*-values
N	2827	737	2090		737	2090	
	Column %				Column %		
Age at the first eligibility for the program							
18–34 years	16	15	16	0.07	15	15	0.61
35–44 years	26	27	26		27	27	
45–54 years	38	41	37		40	42	
≥ 55 years	20	17	21		18	16	
Gender							
Female	30	29	30	0.44	29	29	1.00
Male	70	71	70		71	71	
Race/ethnicity							
Non-Hispanic white	15	14	16	0.09	15	14	0.22
Non-Hispanic black	54	52	54		51	54	
Hispanic	28	31	27		31	28	
Others	3	4	3		3	4	
Education							
Less than a high school diploma	46	44	47	0.14	45	45	0.28
High school diploma or higher	51	53	51		52	53	
Other	3	3	2		3	2	
Current substance use pattern							
Never	74	78	73	0.03	79	75	0.28
Less than weekly or once a week	7	6	8		5	6	
Several times per week	8	8	8		7	8	
Daily	6	5	6		6	7	
Unknown	5	3	5		3	4	
Currently participating in a substance use treatment course	23	25	23	0.27	25	25	1.00
Currently receiving supplemental security income/disability	41	39	41	0.34	41	42	0.64
Past psychiatric hospitalization	31	27	32	0.01	28	29	0.61
Diagnoses with mental illness except for substance use disorders	100	100	100	NE^a^	99	99	1.00
Diagnosed with substance use disorders	52	54	51	0.16	52	53	0.64
Number activities of daily living for which require assistance							
0	74	79	71	<0.01	77	79	0.36
1	15	13	16		14	12	
2+	11	8	13		9	9	
	Mean (Standard deviation)				Mean (Standard deviation)		
Total Medicaid costs during 2 years prior to baseline	$47,284 (64714)	$41,779 (47718)	$49,226 (69632)		$42,225 (59689^b^)	$42,653 (59689^b^)	

*Abbreviations: NE* Not estimable

^a^Not estimable because variance could not be estimated

^b^Pooled standard deviation

Data sources: NYC Department of Homeless Services, NYC Department of Correction, NYC Department of Health and Mental Hygiene, NYC Human Resources Administration’s Customized Assistance Services and HIV/AIDS Services Administration, and New York State Office of Mental Health

The sequence analysis identified 6 distinct patterns (Fig. 1). These represent non-overlapping Medicaid expenditure patterns during the 2 years prior to baseline, including *very low coverage* (15%; 71 placed vs. 361 unplaced persons)*, low user* (10%; 71 placed vs. 221 unplaced persons), *middle user* (17%; 140 placed vs. 341 unplaced persons), *emerging user* (23%; 172 placed vs. 491 unplaced persons)*, second-highest user* (19%; 186 placed vs. 359 unplaced persons)*,* and *high user* (15%; 97 placed vs. 317 unplaced persons).

The *very low coverage* pattern was composed of individuals who were not covered by Medicaid during most of the 2-year period except for later months and had monthly Medicaid costs of $921 on average (3941 on standard deviation, $13 on median, $0 on 25th percentile, $523 on 75th percentile) when they were on Medicaid. These individuals exhibited a pattern of having persistent Medicaid coverage and low Medicaid expenditures (*low user*; $802 on average per month; 4295 on standard deviation, $0 on median, $0 on 25th percentile, $264 on 75th percentile). *Middle user* was composed of individuals who had a consistent pattern of monthly Medicaid costs of $755 on average (2879 on standard deviation, $303 on median, $156 on 25th percentile, $625 on 75th percentile) throughout the 2-year period. There were individuals with almost complete Medicaid coverage who experienced a sharp cost increase after an initial period of not being on Medicaid (*emerging user*). Lastly, there were 2 consistent patterns of high Medicaid expenditures: *high user* with Medicaid costs of $5290 on average per month (7821 on standard deviation, $2892 on median, $879 on 25th percentile, $5954 on 75th percentile), and *second-highest user* with monthly Medicaid costs of $1987 on average (3351 on standard deviation, $1276 on median, $770 on 25th percentile, $2061 on 75th percentile).

In general, each expenditure pattern displayed a unique profile of baseline characteristics that was distinguishable from the others (Table 2). Individuals with patterns of high expenditures were disproportionately more likely to have a history of psychiatric hospitalizations and substance use disorders and at the time of application were more likely to have been participating in a substance use treatment course than those with the other patterns. In addition, those with *very low coverage* and *emerging user* patterns were less likely than others to have been receiving supplemental security at the time of application. Lastly, for more than 80% of the 2-year period prior to baseline, the housing applicants were on Medicaid, except for the *very low coverage* and *emerging user* whose Medicaid coverage was less than 53%.

**Table 2:** Selected baseline demographic, clinical, and service utilization characteristics at the time of application to the New York City supportive housing program by Medicaid expenditure patterns

	Very low coverage	Low user	Middle user	Emerging user	Second-highest user	High user	*p*-values
N: total	432	292	481	663	545	414	
N: placed vs. unplaced	71 vs. 361	71 vs. 221	140 vs. 341	172 vs. 491	186 vs. 359	97 vs. 317	
				Column %			
Age at the first eligibility for the program							
18–34 years	20	9	19	21	11	12	< 0.01
35–44 years	19	23	27	28	25	32	
45–54 years	34	41	32	37	44	43	
≥ 55 years	27	28	21	14	20	13	
Gender							
Female	23	24	37	27	38	28	< 0.01
Male	77	76	63	73	62	72	
Race/ethnicity							
Non-Hispanic white	17	16	14	15	16	11	< 0.01
Non-Hispanic black	58	58	56	51	45	58	
Hispanic	22	22	27	29	36	29	
Others	3	3	3	4	3	2	
Education							
Less than a high school diploma	43	38	45	43	50	56	< 0.01
High school diploma or higher	54	58	53	54	47	43	
Others	3	4	2	3	3	1	
Current substance use pattern							
Never	73	64	77	76	80	67	< 0.01
Less than weekly or once a week	8	8	5	7	6	11	
Several times per week	8	11	7	8	8	9	
Daily	8	11	5	5	4	6	
Unknown	4	5	5	5	2	7	
Currently participating in a substance use treatment course	9	9	11	22	43	36	< 0.01
Past psychiatric hospitalization	28	27	28	30	30	43	< 0.01
Currently receiving supplemental security income/disability	28	51	43	26	53	50	< 0.01
Diagnoses with mental illness except for substance use disorders	100	100	100	100	100	100	0.74
Diagnosed with substance use disorders	48	49	43	48	56	69	< 0.01
Number activities of daily living for which require assistance							
0	70	70	76	78	73	69	< 0.01
1	16	15	12	15	16	17	
2+	14	15	12	7	11	14	
	Mean (standard deviation)						
Total Medicaid costs during 2 years prior to baseline	$6538 (18,831)	$24,504 (39,554)	$20,349 (19,700)	$32,042 (32,975)	$56,683 (34,390)	$149,203 (97,882)	
Specific Medicaid costs during 2 years prior to baseline^a^							
Outpatient care	$799 (2269)	$2039 (3505)	$3120 (3921)	$6134 (8576)	$16,170 (12,137)	$17,255 (18,254)	
Inpatient care	$4583 (17,523)	$18,116 (36,986)	$9014 (17,980)	$17,430 (29,223)	$19,131 (30,993)	$88,800 (92,682)	
Emergency department visits	$134 (360)	$528 (1102)	$531 (1030)	$771 (1219)	$1167 (2410)	$2342 (4782)	
Prescription drugs	$396 (1415)	$1343 (2943)	$2313 (3064)	$3857 (6253)	$11,222 (10,156)	$22,664 (28,724)	
Medicaid coverage days during 2 years prior to baseline	104 (107)	650 (101)	589 (144)	385 (114)	648 (100)	641 (102)	

^a^Sum of specific Medicaid costs did not match up with total Medicaid costs because other Medicaid costs were not reported in this table

Data sources: NYC Department of Homeless Services, NYC Department of Correction, NYC Department of Health and Mental Hygiene, NYC Human Resources Administration’s Customized Assistance Services and HIV/AIDS Services Administration, and New York State Office of Mental Health

**Fig. 2:**
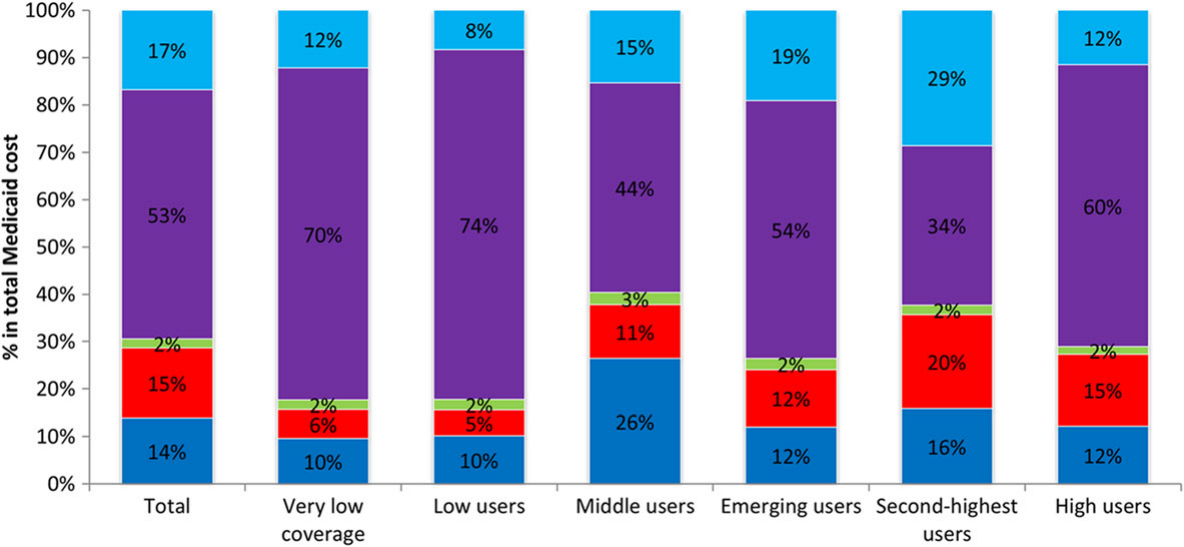
Medicaid cost breakdown by service categories during 2 years prior to baseline by Medicaid expenditure patterns. This figure illustrates how total Medicaid costs were broken down by major service subcategories, stratified by Medicaid expenditure patterns. Outpatient (*light blue*); Inpatient (*purple color*); Emergency (*green color*); prescription (*red color*); other (*blue color*). “Other” mainly consists of Medicaid costs from home health agencies and personal care, managed care capitation, and residential care. NYC Department of Homeless Services, NYC Department of Correction, NYC Department of Health and Mental Hygiene, NYC Human Resources Administration’s Customized Assistance Services and HIV/AIDS Services Administration, and New York State Office of Mental Health

The total mean Medicaid costs over the 2 years prior to baseline was the highest among *high user* ($149,203, 97,882 on standard deviation), followed by *second-highest user* ($56,683, 34,390 on standard deviation) (Table 2). Medicaid utilization among individuals with *very low coverage* and *emerging user* patterns were similar when the contributions of Medicaid subcategories were compared (Fig. 2). Inpatient care constituted the largest proportion of total Medicaid costs (>50%), followed by outpatient care. Medicaid utilization among those with *low user, middle user, second-highest user,* and *high user* patterns were different from those of the previously described *very low coverage* and *emerging user* patterns. Specifically, the percentage of Medicaid costs due to prescription costs (for *high user*) or other expenses (for *low user, middle user*) was greater than that due to outpatient care. Other Medicaid costs for *middle user* were mostly due to managed care costs (73%), whereas those for *low user* were more evenly attributed to three major types of costs (19% managed care, 22% residential care, and 18% home care). Medicaid costs for *second-highest user* were largely attributed to inpatient (34%) and outpatient care types (29%). Emergency department visits contributed the smallest amount of Medicaid costs across all 6 Medicaid expenditure patterns.

Of the 6 expenditure patterns, there were statistically significant cost savings among those with *very low coverage* pattern (−$15,694, 95% CI = −$35,926 to -$7983), *emerging user* (−$9020, 95% CI = −$26,753 to -$1705), and *second-highest user* (−$14,450, 95% CI = −$38,232 to -$4454) after accounting for baseline characteristics via propensity score matching (Table 3). Among all of the expenditure patterns combined, the housing program was also associated with total Medicaid cost savings (−$9526, 95% CI = −$19,038 to -$2003). For *emerging user*, *second-highest user*, and all 6 expenditure patterns combined, these savings represented about a quarter of 2-year Medicaid costs prior to baseline; for individuals with *very low coverage*, the savings were equivalent with almost 240% of baseline costs. Cost savings were mostly driven by a reduction in inpatient services, which represented more than 75% of cost differences between the placed and unplaced individuals. Cost savings from inpatient services were due to cost reductions in psychiatric hospitalizations among placed individuals (Additional file 4). Additionally, some cost savings from emergency department visits were observed among individuals with *second-highest user* and *high user* patterns.

**Table 3:** Weighted mean difference in Medicaid eligible days and Medicaid costs (95% confidence intervals) during 2-year post baseline between placed and unplaced individuals across Medicaid expenditure patterns

	Total	Very low coverage	Low user	Middle user	Emerging user	Second-highest user	High user
N: placed vs. unplaced	737 vs. 2090	71 vs. 361	71 vs. 221	140 vs. 341	172 vs. 491	186 vs. 359	97 vs. 317
Medicaid eligible days							
Placed vs. unplaced	597 vs. 569	344 vs. 343	635 vs. 620	636 vs. 598	585 vs. 541	659 vs. 665	644 vs. 633
Total Medicaid costs							
Difference	-$9526 (−19,038, -2003)	-$15,694 (−35,926, -7983)	-$6477 (−20,529, 17,093)	-$533 (−20,000, 5604)	-$9020 (−26,753, -1705)	-$14,450 (−38,232, -4454)	-$34,100 (−72,238, 24,126)
Medicaid costs due to outpatient care^a^							
Difference	$69 (−1217, 2000)	-$1134 (−2872,1027)	-$704 (−4638, 2717)	$335 (−3453, 3261)	-$1669 (−4446, 906)	$158 (−4662, 3773)	-$1213 (−8043, 2720)
Medicaid costs due to inpatient care^a^							
Difference	-$5864 (−12,251, -123)	-$9365 (−23,376, -4961)	-$1931 (−1,208,116, 758)	-$2485 (−11,164, 4350)	-$7260 (−17,557, -3068)	-$11,099 (−27,596, -3115)	-$26,269 (−54,756, 23,038)
Medicaid costs due to emergency department visits^a^							
Difference	-$318 (−598, −26)	-$130 (−244, 509)	$84 (−250, 962)	-$128 (−394, 845)	-$333 (−898, 573)	-$596 (−3524, -378)	-$1147 (−2262, −443)
Medicaid costs due to prescription drugs^a^							
Difference	-$2014 (−3931, −998)	-$1141 (−3438, 2887)	-$1698 (−4434, 1528)	$380 (−1547, 2731)	-$1662 (−4232, 205)	-$763 (−5929, 2686)	-$4168 (−14,825, 3965)

Data sources: NYC Department of Homeless Services, NYC Department of Correction, NYC Department of Health and Mental Hygiene, NYC Human Resources Administration’s Customized Assistance Services and HIV/AIDS Services Administration, and New York State Office of Mental Health

^a^Sum of specific Medicaid costs did not match up with total Medicaid costs because other Medicaid costs were not reported in this table

Table 4 shows that pre-baseline managed care enrollment was not different between placed and unplaced individuals. After placement, the overall percent of those who were in managed care was greater among placed than unplaced individuals (43% vs. 33%, *p* < 0.05). Stratified by Medicaid expenditure patterns, this difference was statistically significant only among *emerging user* and *second-highest user*.

**Table 4:** Weighted % of placed versus unplaced managed care enrollees during 2-year period before and after baseline across Medicaid expenditure patterns

	Total	Very low coverage	Low user	Middle user	Emerging user	Second-highest user	High user
N: placed vs. unplaced	737 vs. 2090	71 vs. 361	71 vs. 221	140 vs. 341	172 vs. 491	186 vs. 359	97 vs. 317
Prior to baseline	24% vs 28%	0% vs 3%	4% vs 11%	51% vs 58%	18% vs 23%	35% vs 41%	19% vs 26%
Post baseline	43% vs 33%^a^	19% vs 23%	26% vs 20%	46% vs 42%	52% vs 33%^a^	61% vs 46%^a^	58% vs 35%

^a^Difference in % of managed care enrollees between placed and unplaced groups was statistically significant at *p* < .05 according to conditional logistic regression that accounted for propensity score matching

Data sources: NYC Department of Homeless Services, NYC Department of Correction, NYC Department of Health and Mental Hygiene, NYC Human Resources Administration’s Customized Assistance Services and HIV/AIDS Services Administration, and New York State Office of Mental Health

## Discussion

In this evaluation, we found mean Medicaid savings of $9526 (95% CI = −$19,038 to -$2003) for 2 years post-baseline among the program applicants who were placed in the NYC supportive housing program. Stratified by pre-baseline Medicaid expenditure patterns, we observed mean Medicaid savings of $15,694 (95% CI = −$35,926 to -$7983) among those with the *very low coverage* pattern, $9020 (95% CI = −$26,753 to -$1705) among those with the *emerging user* pattern, and $14,450 (95% CI = −$38,232 to -$4454) among those with the *second-highest user* pattern.

Cost savings for those with *very low coverage*, *emerging user*, and *second-highest user* patterns were mostly driven by reduced costs due to psychiatric hospitalizations. Whereas psychiatric inpatient costs more than doubled from pre-baseline to post-baseline among unplaced individuals with the *very low coverage* pattern ($2573 to $5731), slightly decreased among those with the *emerging user* pattern ($7920 to $5767), or were unchanged among those with the *second-highest user* pattern ($6596 to $6338), substantial reductions in psychiatric inpatient costs were observed among placed individuals (*very low coverage*: $940 to $743; *emerging user*: $6700 to $3432; *second-highest user*: $5884 to $4128). This implies that the supportive housing program may be particularly effective in preventing future inpatient events and reducing the length of hospitalization, which in turn contributes to decreased total Medicaid costs. Along with small cost savings from emergency department visits, these inpatient cost savings are consistent with previous findings that supportive housing is associated with a reduction in the number of emergency department visits and hospital admissions among high Medicaid users [26], homeless adults with disabilities [27], and those with chronic medical illness [28].

Another possible explanation for these cost savings was the increased managed care enrollment after placement. Among the applicants overall, placed people were more likely to enroll in managed care than unplaced people during the post-baseline period. Given that high Medicaid costs were likely to have been controlled under the managed care payment system at least for non-behavioral health-related claims, managed care enrollment among the placed group might have contributed to containing Medicaid costs during 2-year post baseline [29]. Similarly, managed care enrollment was much higher among the placed vs. unplaced people with the *emerging user* and *second-highest user* pattern when stratified by Medicaid expenditure patterns, which supported this hypothesis. Future studies with detailed housing service data are warranted to better understand the association between housing placement and increased managed care enrollment. Additionally, estimates of the real cost of managed care beyond capitation costs are needed to explain contributions of managed care enrollment to cost savings.

Despite the fact that this evaluation, like other studies, found an association between living in a supportive housing program and mean Medicaid savings in inpatient and emergency department hospitalizations, the magnitude of the program impact on Medicaid savings in this evaluation is smaller than has been found in previous studies [5, 10, 26, 27]. In addition, although the largest point estimate of Medicaid savings was observed among individuals with the *high user* pattern (−$34,100, 95% CI = −$72,238 to $24,126), this estimate was not statistically significant, indicating high estimation uncertainty. This lack of statistically significant savings may reflect the fact that the high user group had the highest prevalence of pre-existing chronic diseases that would still require treatment after placement. Even if some medical costs for treatment inevitably occur after housing placement, previous studies highlight reduced numbers of emergency department and hospital admission events instead of evaluating total medical costs [28]. The difference in findings may be also due to the small sample size [5, 28] or lack of appropriate control groups in previous studies [10].

This evaluation has some limitations. First, because applicants were not randomly selected for housing placement, our results might be biased due to unobserved confounders. Despite thorough efforts to ensure comparability between placed and unplaced groups via propensity score matching and sequence analysis, two groups could be different in unobserved characteristics, which could affect Medicaid utilization post-baseline. Second, Medicaid expenditure patterns were identified based on the assumption that there were underlying groups of individuals who have distinct trajectories of Medicaid utilization. This assumption is consistent with health policy approaches of distinguishing high Medicaid users from low users [2, 4, 30]. However, clustering from sequence analysis has been criticized because it has been difficult to replicate using a population other than the one studied in any given analysis [31]. Third, Medicaid costs from managed care capitations were classified as costs due to “other” utilization. Although Medicaid expenditure patterns were based on total Medicaid costs and all behavioral health-related claims were carved out and reimbursed via a fee-for-service mechanism during the evaluation period, Medicaid savings due to specific services may be under- or over-estimated. Fourth, even if we accounted for pre-baseline Medicaid coverage, post-baseline coverage might be different between the placed and unplaced groups, which could introduce bias in the observed cost savings. To address this concern, we calculated the average Medicaid coverage days post baseline in each expenditure pattern and found that differences in the length of Medicaid coverage post-baseline between the 2 groups were very small during the full 2-year period, ranging from 5 to 48 days. Fifth, our finding might be biased because the index date was different for the two groups (the first move-in date for the placed group, the first eligibility date for the unplaced group). For placed persons, there was median gap of 95 days between eligibility and housing placement. However, even if substantial changes in Medicaid utilization were unlikely to occur during this period, we included Medicaid costs due to 5 service types as covariates in the propensity score models in order to minimize bias. Lastly, we assumed that housing placement status and confounders were not time-varying. Although we captured anyone placed in the supportive housing program and included them in the placed group, an inability to account for a dynamic aspect of housing and confounding could result in bias.

A main strength of this evaluation is that sequence analysis identified distinct groups of individuals based on 2 years of pre-baseline Medicaid records. Compared with conventional mean- or frequency-based methods, our method produced groups that likely more validly captured patterns of Medicaid utilization because they preserved sequences of claims over a sufficiently long time period [14, 15, 18]. Another strength is that this analysis controlled for confounding using a large number of variables that measure baseline demographic and behavioral characteristics. Lastly, the use of comparison groups and the larger sample size relative to that in previous studies strengthened the internal validity of the findings.

## Conclusion

In conclusion, in an NYC supportive housing program for people with serious mental illness and chronic homelessness or dual diagnoses of mental illness and substance use disorder, there were Medicaid savings overall as well as for tenants who had patterns of *very low coverage*, *emerging user*, or *second-highest user* of Medicaid-funded services prior to baseline. This evaluation found that tenancy in a supportive housing program among individuals at risk of homelessness and with serious mental illness had a significant impact on Medicaid expenditures, and that cost savings were mostly driven by reduced psychiatric hospitalizations. These findings call for further evaluation of this program to examine its long-term impact and inclusion of detailed data about housing services or referrals to identify mechanisms through which supportive housing placement influences Medicaid cost savings.

## Additional files

Additional file 1: A flow chart of sample selection process. This file illustrates how exclusion criteria were applied in order to generate a final evaluation sample. (DOCX 20 kb)

Additional file 2: Covariates included in the propensity score models. This file contains a list of covariates that were included in the propensity score models. (DOCX 19 kb)

Additional file 3: Summary of balance of baseline characteristics between placed and unplaced people before and after propensity score matching. This file shows performance of propensity score matching in balancing baseline characteristics between placed and unplaced people. (DOCX 13 kb)

Additional file 4: Weighted mean psychiatric inpatient costs among placed versus unplaced persons with very low coverage, emerging user, and second-highest user patterns. This file shows weighted mean psychiatric inpatient costs among placed and unplaced persons, stratified by three trajectory groups, including very low coverage, emerging user, and second-highest user patterns. (DOCX 12 kb)

## Abbreviations

NYC: New York City
US: United States
