# Supplementary material for: Impact of a New York City supportive housing program on Medicaid expenditure patterns among people with serious mental illness and chronic homelessness
Source: BMC Health Serv Res. 2018 Jan 10;18:15. doi: 10.1186/s12913-017-2816-9 (PMC5761184; doi:10.1186/s12913-017-2816-9)
Supplement: Supplementary file 1 — A flow chart of sample selection process. This file illustrates how exclusion criteria were applied in order to generate a final evaluation sample. (DOCX 20 kb) [file 12913_2017_2816_MOESM1_ESM.docx]

A flow chart of sample selection process

3,922 applicants with 2-year follow-up time

757 placed persons

3,165 unplaced persons (including 3 persons placed for ≤ 7 days)

737 placed persons

20 placed persons with continuous housing experiences

2,090 unplaced persons

1,075 unplaced persons with other housing placement during the first 6 months
