# Supplementary material for: Impact of a New York City supportive housing program on Medicaid expenditure patterns among people with serious mental illness and chronic homelessness
Source: BMC Health Serv Res. 2018 Jan 10;18:15. doi: 10.1186/s12913-017-2816-9 (PMC5761184; doi:10.1186/s12913-017-2816-9)
Supplement: Supplementary file 2 — Covariates included in the propensity score models. This file contains a list of covariates that were included in the propensity score models. (DOCX 19 kb) [file 12913_2017_2816_MOESM2_ESM.docx]

Covariates included in the propensity score models

|  | Covariates | Total | Very low coverage | Low user | Middle user | Emerging user | Second-highest user | High user |
| --- | --- | --- | --- | --- | --- | --- | --- | --- |
| Baseline demographic characteristics | Age at the earliest eligibility for the program (categories) | *X* | *X* | *X* | *X* | *X* | *X* | *X* |
|  | Sex | *X* | *X* | *X* | *X* | *X* | *X* | *X* |
|  | Race/ethnicity | *X* | *X* | *X* | *X* | *X* | *X* | *X* |
|  | Education | *X* | *X* | *X* | *X* | *X* | *X* | *X* |
|  | Citizenship | *X* | *X* | *X* | *X* | *X* | *X* | *X* |
|  | Language | *X* | *X* | *X* | *X* | *X* | *X* | *X* |
|  | Veteran status | *X* | *X* | *X* | *X* | *X* | *X* | *X* |
| Baseline substance use characteristics | Current substance use pattern | *X* | *X* | *X* | *X* | *X* | *X* |  |
|  | Past substance use pattern | *X* | *X* | *X* | *X* | *X* | *X* | *X* |
|  | Currently participating in substance use program | *X* | *X* | *X* | *X* | *X* | *X* | *X* |
|  | Completed substance use program | *X* | *X* | *X* | *X* | *X* | *X* | *X* |
|  | Diagnosed with substance use disorders | *X* | *X* | *X* | *X* | *X* | *X* | *X* |
| Baseline mental health characteristics | Diagnosed with mental illness or substance use disorders |  |  |  |  |  |  |  |
|  | Has mental retardation | *X* | *X* |  | *X* | *X* | *X* |  |
|  | Has any mental illness other than mental retardation | *X* |  |  | *X* | *X* | *X* |  |
|  | History of mental health-related symptoms/behaviors | *X* | *X* | *X* | *X* | *X* | *X* | *X* |
|  | Current mental health-related symptoms/behaviors | *X* | *X* | *X* | *X* | *X* | *X* | *X* |
|  | History of violent symptoms/behaviors | *X* | *X* | *X* | *X* | *X* | *X* | *X* |
|  | Current violent symptoms/behaviors | *X* |  | *X* |  |  |  |  |
|  | Past psychiatric hospitalization | *X* | *X* | *X* | *X* | *X* | *X* | *X* |
| Baseline physical health characteristics | Any diagnosis of physical illness |  |  |  |  |  |  |  |
|  | Number of types of physical illness diagnoses (categories) | *X* |  |  |  |  | *X* | *X* |
|  | Any severe physical illness diagnosis according to Charlson comorbidity index^†^ |  |  |  |  |  |  |  |
|  | Number of severe physical illness diagnoses (categories) | *X* | *X* |  |  | *X* |  |  |
|  | Comorbidity of mental and physical diagnoses | *X* | *X* | *X* | *X* | *X* | *X* | *X* |
|  | Comorbidity of mental and severe physical diagnoses | *X* | *X* | *X* | *X* | *X* | *X* | *X* |
|  | Number of activities of daily living that require assistance (categories) | *X* |  |  | *X* | *X* | *X* | *X* |
|  | Currently hospitalized | *X* | *X* |  | *X* | *X* | *X* | *X* |
|  | Currently hospitalized, incarcerated, in foster care, or in other institution | *X* | *X* |  | *X* | *X* | *X* |  |
| Housing eligibility information | Approved for single-site housing | *X* | *X* | *X* | *X* | *X* | *X* |  |
|  | Approved for scattered site housing | *X* | *X* | *X* | *X* | *X* | *X* | *X* |
|  | Recommended for 24-hour supervision | *X* |  |  |  | *X* |  | *X* |
|  | Recommended for assisted outpatient treatment | *X* |  | *X* |  | *X* |  | *X* |
|  | Recommended for case management | *X* | *X* | *X* | *X* | *X* | *X* | *X* |
|  | Recommended for child care | *X* |  |  |  |  |  |  |
|  | Recommended for domestic violence services | *X* |  |  | *X* | *X* | *X* |  |
|  | Recommended for financial management services | *X* | *X* | *X* | *X* | *X* | *X* | *X* |
|  | Recommended for primary health care | *X* | *X* | *X* | *X* | *X* | *X* | *X* |
|  | Recommended for vocational/educational training program | *X* | *X* | *X* | *X* | *X* | *X* | *X* |
|  | Recommended for medical treatment | *X* | *X* | *X* | *X* | *X* | *X* | *X* |
|  | Recommended for medication management | *X* | *X* | *X* | *X* | *X* | *X* | *X* |
|  | Recommended for mental health treatment | *X* | *X* | *X* | *X* | *X* | *X* | *X* |
|  | Recommended for Metal illness and Chemical Addition program | *X* | *X* | *X* | *X* | *X* | *X* | *X* |
|  | Recommended for parenting skills training | *X* |  |  |  |  |  |  |
|  | Recommended for psychiatric treatment | *X* |  |  |  | *X* |  |  |
|  | Recommended for substance use treatment | *X* | *X* | *X* | *X* | *X* | *X* | *X* |
| Receipt of benefits at baseline | Social security | *X* | *X* | *X* | *X* | *X* | *X* | *X* |
|  | Supplementary Security Income | *X* | *X* | *X* | *X* | *X* | *X* | *X* |
|  | HIV/AIDS Services Administration | *X* | *X* | *X* | *X* | *X* | *X* | *X* |
|  | Pension | *X* |  |  | *X* | *X* | *X* |  |
|  | Medicare | *X* | *X* | *X* | *X* | *X* | *X* | *X* |
|  | Veteran's benefits | *X* | *X* | *X* | *X* | *X* | *X* | *X* |
| Amount of services/benefits received during 2 years prior to baseline | Cost of New York City single adult homeless^‡^ shelters | *X* | *X* | *X* | *X* | *X* | *X* | *X* |
|  | Cost of New York City family homeless^‡^ shelters | *X* | *X* | *X* | *X* | *X* | *X* | *X* |
|  | Cost of New York City jails^‡^ | *X* | *X* | *X* | *X* | *X* | *X* | *X* |
|  | Costs of Medicaid emergency department visits^‡^ | *X* | *X* | *X* | *X* | *X* | *X* | *X* |
|  | Costs of Medicaid inpatient hospitalizations^‡^ | *X* | *X* | *X* | *X* | *X* | *X* | *X* |
|  | Costs of Medicaid outpatient visits^‡^ | *X* | *X* | *X* | *X* | *X* | *X* | *X* |
|  | Costs of Medicaid-reimbursed prescriptions^‡^ | *X* | *X* | *X* | *X* | *X* | *X* | *X* |
|  | Costs of Medicaid due to other reasons^‡^ | *X* | *X* | *X* | *X* | *X* | *X* | *X* |
|  | Costs from food stamps^‡^ | *X* | *X* | *X* | *X* | *X* | *X* | *X* |
|  | Total services/benefits costs (Medicaid, homeless shelters, jails, state-operated psychiatric centers, food stamps, government subsidized housing) ^‡^ | *X* | *X* | *X* | *X* | *X* | *X* | *X* |

^†^Charlson ME, Pompei P, Ales KL, MacKenzie CR. A new method of classifying prognostic comorbidity in longitudinal studies: development and validation. J Chronic Dis. 1987,40(5):373-383.

^‡^continuous variables.

Data sources: NYC Department of Homeless Services, NYC Department of Correction, NYC Department of Health and Mental Hygiene, NYC Human Resources Administration’s Customized Assistance Services and HIV/AIDS Services Administration, and New York State Office of Mental Health.
