# Supplementary material for: Impact of a New York City supportive housing program on Medicaid expenditure patterns among people with serious mental illness and chronic homelessness
Source: BMC Health Serv Res. 2018 Jan 10;18:15. doi: 10.1186/s12913-017-2816-9 (PMC5761184; doi:10.1186/s12913-017-2816-9)
Supplement: Supplementary file 3 — Summary of balance of baseline characteristics between placed and unplaced people before and after propensity score matching. This file shows performance of propensity score matching in balancing baseline characteristics between placed and unplaced people. (DOCX 13 kb) [file 12913_2017_2816_MOESM3_ESM.docx]

Summary of balance of baseline characteristics between placed and unplaced people before and after propensity score matching

|  | Total | Very low coverage | Low user | Middle user | Emerging user | Second-highest user | High user | |
| --- | --- | --- | --- | --- | --- | --- | --- | --- |
| Total number of covariates† | 101 | 79 | 73 | 81 | 88 | 88 | 78 |  |
| % of covariates† with no change or decreased imbalances‡ | 81% | 66% | 63% | 64% | 69% | 59% | 74% |  |
|  | Mean standardized absolute difference (before matching vs. after matching) | | | | | | |  |
| Covariates† with increased imbalances§ | 0.024 vs. 0.051 | 0.057 vs. 0.130 | 0.061 vs. 0.138 | 0.048 vs. 0.107 | 0.034 vs. 0.065 | 0.040 vs. 0.104 | 0.068 vs. 0.120 |  |
| Covariates† with no change or decreased imbalances‡ | 0.098 vs. 0.031 | 0.185 vs. 0.052 | 0.213 vs. 0.071 | 0.135 vs. 0.052 | 0.129 vs. 0.031 | 0.128 vs. 0.051 | 0.221 vs. 0.082 |  |
| All covariates† | 0.084 vs. 0.035 | 0.141 vs. 0.078 | 0.157 vs. 0.096 | 0.104 vs. 0.072 | 0.100 vs. 0.041 | 0.092 vs. 0.073 | 0.182 vs. 0.092 |  |

Abbreviations: before = before propensity score matching, after = after propensity score matching.

†included response categories of categorical variables.

‡covariates that had baseline difference between placed and unplaced groups unchanged or smaller after propensity score matching.

§covariates that had baseline difference between placed and unplaced groups larger after propensity score matching.

Data sources: NYC Department of Homeless Services, NYC Department of Correction, NYC Department of Health and Mental Hygiene, NYC Human Resources Administration’s Customized Assistance Services and HIV/AIDS Services Administration, and New York State Office of Mental Health.
