# Supplementary material for: Impact of a New York City supportive housing program on Medicaid expenditure patterns among people with serious mental illness and chronic homelessness
Source: BMC Health Serv Res. 2018 Jan 10;18:15. doi: 10.1186/s12913-017-2816-9 (PMC5761184; doi:10.1186/s12913-017-2816-9)
Supplement: Supplementary file 4 — Weighted mean psychiatric inpatient costs among placed versus unplaced persons with very low coverage, emerging user, and second-highest user patterns. This file shows weighted mean psychiatric inpatient costs among placed and unplaced persons, stratified by three trajectory groups, including very low coverage, emerging user, and second-highest user patterns. (DOCX 12 kb) [file 12913_2017_2816_MOESM4_ESM.docx]

Weighted mean psychiatric inpatient costs among placed versus unplaced persons with very low coverage, emerging user, and second-highest user patterns

|  | Very low coverage | Emerging user | Second-highest user |
| --- | --- | --- | --- |
| Prior to baseline | $940 vs. $2,573 | $6,700 vs. $7,920 | $5,783 vs. $6,596 |
| Post baseline | $743 vs. $5,731 | $3,432 vs. $5,767 | $4,073 vs. $6,338 |

Data sources: NYC Department of Homeless Services, NYC Department of Correction, NYC Department of Health and Mental Hygiene, NYC Human Resources Administration’s Customized Assistance Services and HIV/AIDS Services Administration, and New York State Office of Mental Health.
